# The role of access to electricity, female education, and public health expenditure on female health outcomes: evidence from SAARC-ASEAN countries

**DOI:** 10.1186/s12905-021-01520-0

**Published:** 2021-11-01

**Authors:** Mohammad Mafizur Rahman, Khosrul Alam

**Affiliations:** 1 School of Business, University of Southern Queensland, Toowoomba, QLD 4350 Australia; 2 Department of Economics, Bangabandhu Sheikh Mujibur Rahman Science and Technology University, Gopalganj, 8100 Bangladesh

**Keywords:** Access to electricity, Female education, Public health expenditure, Female life expectancy, Female adult mortality, I10, I15, I18, C23

## Abstract

**Background:**

The importance of the status of female health should have research priority due to the unique medical needs of women. Hence this paper attempts to explore the nexus of access to electricity, female education, and public health expenditure with female health outcomes in the SAARC-ASEAN countries.

**Methods:**

Using the data of 2002–2018, and applying the cross-sectional dependence test, Modified Wald test, Wooldridge test, the Panel corrected standard error (PCSE) model, the Feasible generalized least square (FGLS) model, and the pair-wise Granger causality test, the robust outcomes on female health are found.

**Results:**

Access to electricity, female education rate, public health expenditure, economic growth, and immunization rate, all have a positive effect on female life expectancy at birth, and a negative effect on the female adult mortality rate. The urbanization rate has a significantly positive impact on female life expectancy at birth but an insignificant impact on female adult mortality rate. The one-way causal relationship between the variables are also revealed.

**Conclusions:**

All the results are rational and have important milestone for the health sector. The health status of females should be improved and protected by formulating effective policies on access to electricity, female education, public health expenditure, immunization, economic growth, and urbanization.

## Introduction

The importance of the status of female health cannot be overlooked and should be prioritized in order to balance gender equality and fulfil the unique medical needs of women. Female health status is often ignored, especially in developing countries, due to the disadvantaged conditions they face, created by discrimination which is deeply rooted in biological, socio-economic, cultural, political, and spiritual grounds [1, 2]. To end this underprivileged situation, the United Nations Development Program (UNDP) focused on the sustainable development goals to reduce gender inequality and ensure better health facilities for females [3, 4]. Despite numerous efforts to ensure more facilities, the health care access of females still remains a greater challenge in the contemporary world. Thus further research for identifying contributory factors to female health outcomes is quite appealing and crucial for policy makers.

Against this backdrop, our present study is an endeavour to probe the determining factors of female health in the selected 10 SAARC-ASEAN countries.^1^ Having a GDP of US$ 436.491 billion, these countries belong to the group of those having a total population of 2.007 billion, out of whom 48.626% are female [5]. The average life expectancy of females in these countries in 2018 was 73.985 years, where the highest life expectancy was in Thailand (80.704 years) and lowest was in Pakistan (68.109 years) [5]. These figures are lower than some other regions of the world such as EU and North America where average female life expectancy of these two regions are 83.867 years and 81.405 years, respectively [5]. In 2018, the average female adult mortality rate in these countries was 126.411 per 1,000 female adults (ages 15–60 years), where the highest mortality is experienced by Bhutan (194.721 per 1000 female adults) and the lowest mortality was in Sri Lanka (56.065 per 1000 female adults) [5]. This is higher than some other regions of the world such as EU and North America where the average female adult mortality of these two regions was 52.251 and 81.935, respectively [5]. The average rate of access to electricity, female education, immunization, and urbanization in these countries in 2018 were 90.611%, 90.500%, 39.150%, respectively [5]. The average per capita public health expenditure in this region is US$119.279 [5]. All these indicators need more careful attention for ensuring better health outcomes of female.

Some empirical studies can be found in the literature [6–45, 61, 62, 72] that has endeavoured to uncover the determining elements of the health outcomes of people where inclusive health issues are rarely addressed. Female health outcomes have been analysed by some studies, but those studies failed to include some important factors like access to electricity, female education rate, immunization rate, economic growth rate, urbanization rate, and public health expenditure, all of which have important policy implications. Thus our main aim is to fill up the prevailing literary gaps in this current study, where we will thoroughly probe the effects of access to electricity, female education rate, public health expenditure, immunization rate, economic growth rate, and urbanization rate on female health outcomes in the SAARC-ASEAN countries.

The rationale for selecting the variables is that: (i) access to electricity ensures better health outcomes for females by providing health related facilities, like getting more health related knowledge from TV, Radio, etc. and become more health conscious, and receiving more medical benefits from electricity run appliances; (ii) female education creates awareness and provides proper guidelines about the healthy life to ensure better health for females; (iii) public health expenditure provides various medical facilities at a lower cost for the improvement of female health. (iv) economic growth ensures better living standards that facilitate improved female health; (v) a higher immunization rate generates protection against infectious diseases and safeguards female health; and (vi) urbanization creates different modern facilities that may positively affect female health. More rationalization of the selection of variables is provided in Sect. 3.1.

The major objectives of this study are:

(i) To identify the impact of access to electricity, female education, public health expenditure economic growth, immunization rate, and urbanization on female health outcomes in the SAARC-ASEAN countries.
(ii) To detect the causality between access to electricity, female education, public health expenditure, economic growth, immunization rate, urbanization, and female health outcomes.

The main contributions of this study are: (i) this is the first study in the literature, to the best of our knowledge, that identifies the impact of access to electricity, female education, public health expenditure, economic growth, immunization rate, and urbanization on female health outcomes in the context of SAARC-ASEAN countries; (ii) this study utilizes the updated available and inclusive data considering the period of 17 years (2002–2018); (iii) the outcomes are achieved by using robust econometric tools: cross-sectional dependence test, Modified Wald test, Wooldridge test, the Panel corrected standard error (PCSE) model, the Feasible generalized least square (FGLS) model, and the pair-wise Granger causality test; and (vi) the results will provide unique guidelines for policy makers to advance the improved health status of females by considering access to electricity, female education, public health expenditure, economic growth, immunization rate, and urbanization policies.

The study is aligned in the following order: following the introduction, Sect. 2 reviews the past literature; Sect. 3 describes the methods; Sect. 4 presents s the results; Sect. 5 discusses the results; and Sect. 6 displays the conclusion and policy implications.

## Literature review

We will discuss the related literature concerning the linkages between our considered variables and health outcomes.

### Access to electricity and female health outcomes nexus

Electricity is a vital element of modern society and has a significant impact on the availability and effectiveness of health facilities, as explained in existing literature [6, 7, 11–14]. Wang [6] established that access to electricity, as the mortality variable in urban areas, significantly reduces child mortality in 60 low-income countries. Adair-Rohani et al. [11] provided a specific example, and identified that electricity access plays significantly positive role on health in the case of 11 sub-Saharan African countries. Irwin et al. [12] pointed out that access to electricity has a positive influence on health outcomes, for example lower mortality, less susceptibility to diseases, and improved health care, whereas poor access to electricity increases those risks and leads to poorer quality health services. Chen et al. [7] found that rural electrification programs improved primary health care facilities by increasing the utilization of health services, receiving vaccinations by children, and receiving maternal antenatal care because of the availability and functionality of essential medical devices and health information is easily received through electronic media in case of Gujarat, India. In addition, Bridge et al. [13] confirmed the positive but insignificant effect of access to electricity on health in Nepal for the data period of 2010–2011. Hernández [14] observed that energy/electricity insecurity led to adverse health consequences in the USA.

### Female education rate and female health outcomes nexus

Because education increases consciousness about important health issues, it is also considered to be an important element for improving health outcomes. The significant role of education on health outcomes is shown by many contemporary researchers like [8, 15–22]. These researchers have focused on a range of disparate countries. Furnee et al. [15] explored the positive effects of education on the health status of both males and females in the US and Europe by using the meta-analysis approach. Similarly, Keats [19] found that female schooling had a positive impact on child health, using Uganda's free primary education program of 1997 as a case study. McAlister and Baskett [16] surveyed 148 countries and observed that female education had the greatest effect on maternal mortality. Anlimachie and Avoada [17] cited the rural education program in Ghana, which had improved the social and health wellbeing of the citizens. Rahman and Alam [8] used the data of 1975–2019 and employed ARDL bounds test and pair-wise Granger causality analysis and ascertained that female education significantly reduced the child mortality rate in case of Bangladesh. Hurt et al. [18] established that females with at least minimum education had a lower mortality rate than those with no education in rural Bangladesh. Kanmiki et al. [20] identified that the children of mothers who had attained primary or junior high school education were 45% less likely to experience under-five mortality than those whose mothers had no education in rural northern Ghana. Similar findings were also observed by Chowdhury et al. [21] in the case of rural Bangladesh. Akinkugbe and Mohanoe [22] found that in Lesotho, female literacy had a significant determining effect on the life expectancy, infants and under-five mortality.

### Public health expenditure and female health outcomes nexus

Public health expenditure has a significant role in improving health outcomes globally, as found in current literature (see [9, 10, 23, 24, 26–31]). Novignon and Lawanson [10] ascertained that public health expenditure had a positive and significant effect on child health in the case of 45 Sub-Saharan African countries during the period of 1995–2011. Nicholas et al. [23] found that the public health expenditure was inversely and significantly related to infant and under-five mortalities, but had a negative and significant effect on maternal mortality in 40 sub-Saharan African countries during the period of 2000–2010. Farag et al. [26] outlined that government health spending had a significant effect in reducing infant and child mortality in case of 133 low and middle-income countries in the world. Similar results were also observed by Boachie and Ramu [27] for Ghana. Ahmad and Hasan [24] established that public health expenditure had a positive effect on health outcomes in Malaysia. Sango-Coker and Bein [28] obtained that the female population lived longer than male and public health expenditure had positive impact on it in case of West African countries during the period of 1999–2014. Duba et al. [29] found that there was a statistically significant association between life expectancies of both men and women and health care expenditures in the case of 210 countries during 1995–2014. Rahman et al. [9] found that the public health expenditure had a significant impact on reducing the death rate and infant mortality rate but an insignificant impact on life expectancy in case of SAARC-ASEAN regions. Akinkugbe and Mohanoe [22] showed that public health expenditure had a significant effect on the life expectancy, infant and under-5 mortality in Lesotho. Similar identification was also revealed by Makochekanwa and Madziwa [30] for Zimbabwe, and Behera and Dash [72] for South-East Asia region. In contrast, Zaman et al. [31] found no significant relationship between total health expenditure and increased life expectancy in Bangladesh.

### Immunization rate and female health outcomes nexus

Immunization creates protection against various infectious diseases and therefore ensures a healthier life for the people [22, 32–34, 61]. Pezzotti et al. [32] found that universal vaccination programs effectively reduced the preventive diseases of children in Italy where the data of 1900–2015 were used. Applying a dynamic mathematical model of VZV transmission, Brisson et al. [33] showed that the prevalence and morbidity of varicella decreased by the mass vaccination of 12-month-old children. Owais et al. [34] found that immunization had a significant positive impact on child health in the case of Karachi, Pakistan in 2008 where the Poisson regression model was used. Rodrigues and Plotkin [61] identified that an increased immunization rate reduced the number of people suffering from specific diseases and played a role in increasing life expectancy in the USA in 2017. Akinkugbe and Mohanoe [22] ascertained that the child immunization rate has a significant effect on life expectancy, infant and under-5 mortality in Lesotho.

### Economic growth and female health outcomes nexus

Economic growth ensures different health related advanced facilities and medical amenities that increase the health outcomes of people as observed in various works like [8, 9, 22, 25, 35–38]. Wang et al. [25] found that economic growth was positively linked with life expectancy in Pakistan by employing ARDL bounds test approach. Similar findings were also observed by Mahyar [35] for Iran, Ebenstein et al. [37] for China, and Shahbaz et al. [36] for 16 sub-Saharan African countries. Rahman et al. [9] found that economic growth had a significant impact on increasing life expectancy and reducing the infant mortality rate but no significant impact on death rate in the case of 15 SAARC-ASEAN countries that employed fixed effects, random effects and GMM models. Houweling et al. [65] identified that economic growth was associated with a reduction in under-5 mortality in the case of 43 developing countries. Rahman and Alam [8] also established that economic growth led to a significantly negative impact on the child mortality rate in Bangladesh. Wang [38] discerned the importance of economic development for ensuring maternity care in 137 developing countries. Harttgen et al. [66] showed that an increase of economic growth reduced children’s underweight and under nutrition rate, both of which improved child health in sub-Saharan Africa. A similar finding was also revealed by Rashad and Sharaf [64] for Egypt. Gupta and Mitra [67] found that economic growth and health status were positively correlated and had a two‐way relationship from the panel data of Indian states. On the other hand, Granados and Ionides [63] found that in the case of Sweden, the impact of economy on health was significant at lag 0 and 2 in the nineteenth and twentieth century, respectively but no evidence was explored for economic effects on mortality at larger lags. Similarly, Akinkugbe and Mohanoe [22] obtained no significant impact of economic growth on health outcomes in Lesotho.

### Urbanization rate and female health outcomes nexus

Urbanization has a mixed effect on health status by ensuring different modern medical facilities as well as creating negative penalties for the public [8, 39–45, 62]. Wang [39] revealed that the urbanization was positively related to global health outcome by reducing mortality, under-five mortality, and infant mortality and increasing life expectancy in 163 countries. Amouzou et al. [40] found a negative association between urbanization and under-5 mortality in Sub-Saharan Africa. Panahi and Aleemran [41] also found the positive effect of urbanization on life expectancy in Middle Eastern and North African (MENA) Countries. Eckert and Kohler [43] also established a positive but insignificant association between urbanization and life expectancy in developing countries. In contrast, Antai and Moradi [44] found that under-5 mortality steadily increased as urbanization increased during 1983–2003 in Nigeria. Yang et al. [45] found that most of the indicators of urbanization showed that it had a detrimental effect on female health outcomes in China. Adediran et al. [42] found that urbanization significantly increased the frequency of metabolic syndrome (MetS) as an important cause of morbidity and mortality in Nigeria. Torres et al. [62] ascertained that due to urban penalty urbanization created high mortality and lower life expectancy in case of Scotland during the period of 1861–1910. Recently Rahman and Alam [8] also revealed a significant positive association between urbanization and child mortality rate in Bangladesh.

From the critical investigation of the aforementioned literature it can be observed that the previous findings are not sufficiently convincing and encouraging to formulate effective policies, particularly for female health. Moreover, unanimous, complete and comprehensive policy outcomes focusing exclusively on female health status are absent, especially in the SAARC-ASEAN countries. This is the main gap in the existing literature, and our current aim is to fill up this gap and provide uniform and inclusive policies to ensure better health for the female, which will be a milestone for the modern health sector.

## Methods

### Theoretical or empirical rationale for choosing the variables

The theoretical justification for conducting this study is based on the different well-known and well-recognized theories and models, for example the human capital theory by Becker [68], the health care model by Grossman [69], and the neoclassical model by Romer [70].

The rationale of choosing our studied variables is relies on the availability of data, from contemporary and previous literary works. We have considered the variables relating to life expectancy at birth, following Rahman et al. [9], Shahbaz et al. [36], Rodrigues and Plotkin [61], among others; mortality rate following Rahman et al. [9], Hurt et al. [18], Nicholas et al. [23], among others; access to electricity in line with Wang [6], Chen et al. [7], Bridge et al. [13], among others; female education rate following [8], Hurt et al. [18], Keats [19]; public health expenditure in line with Rahman et al. [9], Novignon and Lawanson [10], Nicholas et al. [23], among others; immunization rate following Akinkugbe and Mohanoe [22], Owais et al. [34], Rodrigues and Plotkin [61], among others; urbanization rate in line with Rahman and Alam [8], Wang [39], Amouzou et al. [40], among others; and economic growth corresponding to the research undertaken by Rahman and Alam [8], Rahman et al. [9], Wang et al. [25], among others.

### Variables and data

For this study the female health outcomes are considered as the dependent variables; for this, female life expectancy (FLE) at birth and female adult mortality rate (FAM) per 1,000 female adults (ages 15–60 years) are used as proxy variables. Along with these, the access to electricity (AEL), female education rate, immunization rate (IMM), urbanization rate (URB), gross domestic product (GDP) and public health expenditures (PUH) are taken as the independent variables. Access to electricity is the percentage of population having access to electricity; female education rate is the school enrolment of females at secondary level as a percentage of gross; immunization rate is taken as measles vaccination taking rate as the percentage of children ages 12–23 months; GDP is used to see the reflection of economic growth; urbanization is defined as the urban population referring to people living in urban areas as a percentage of total population; and the public health expenditure is taken as the domestic general health expenditure per capita at current US$ funded by the government.

All the data are collected from the World Development Indicator [5] of the World Bank over the years 2002–2018. Some missing data of female school enrolment at secondary level are linearly interpolated through E-views-11. To accomplish the estimation, we have used two well-known statistical software packages, STATA-16 and E-views1.

The models used for the estimation of this study are presented below:

1 $${\text{FLE }} = {\text{ f }}\left( {{\text{AEL}},{\text{ FED}},{\text{ PUH}},{\text{ GDP}},{\text{ IMM}},{\text{ URB}}} \right)$$

2 $${\text{FAM }} = {\text{ f }}\left( {{\text{AEL}},{\text{ FED}},{\text{ PUH}},{\text{ GDP}},{\text{ IMM}},{\text{ URB}}} \right)$$

To get the direct elasticity of each variable from the coefficients, we have transformed all the variables of the Eqs. (1) and (2) into natural logarithmic form. Thus, the Eqs. (1) and (2) can be written as:

3 $${\mathrm{LNFLE}}_{\mathrm{t}}=\mathrm{\alpha }+{\upbeta }_{1}{\mathrm{LNAEL}}_{\mathrm{t}}+{\upbeta }_{2}{\mathrm{LNFED}}_{\mathrm{t}}+{\upbeta }_{3}{\mathrm{LNPUH}}_{\mathrm{t}}+{\upbeta }_{4}{\mathrm{LNGDP}}_{\mathrm{t}}+{\upbeta }_{5}{\mathrm{LNIMM}}_{\mathrm{t}}+{\upbeta }_{6}{\mathrm{LNURB}}_{\mathrm{t}}+{\upvarepsilon }_{\mathrm{t}}$$

4 $${\mathrm{LNFAM}}_{\mathrm{t}}=\mathrm{\alpha }+{\upbeta }_{1}{\mathrm{LNAEL}}_{\mathrm{t}}+{\upbeta }_{2}{\mathrm{LNFED}}_{\mathrm{t}}+{\upbeta }_{3}{\mathrm{LNPUH}}_{\mathrm{t}}+{\upbeta }_{4}{\mathrm{LNGDP}}_{\mathrm{t}}+{\upbeta }_{5}{\mathrm{LNIMM}}_{\mathrm{t}}+{\upbeta }_{6}{\mathrm{LNURB}}_{\mathrm{t}}+{\upvarepsilon }_{\mathrm{t}}$$

where, α is the intercept, and β_1_, β_2_, β_3_, β_4_, β_5_, β_6_ are coefficients and ε_t_ is the error term.

### Econometric approach

For empirical estimation we employed a number of renowned econometric approaches. We conducted the mentioned tests as: a cross-sectional dependence test to identify the shock effect; the Modified Wald test for group wise heteroskedasticity and Wooldridge test for aut-1ocorrelation in panel data to observe the heteroskedasticity and autocorrelation respectively. We employed the Panel corrected standard error (PCSE) model and the Feasible generalized least square (FGLS) model to obtain results that will show the robust relations between the variables; and the pair-wise Granger causality to determine the direction of causality.

Because of the resemblance of the geographic, economic, historical, ethnic and political shocks, the cross-sectional dependence of the variables may be observed. In this study we have employed four well-known cross-sectional dependency tests: Breusch and Pagan [46] BP LM, Pesaran [47] scaled LM, Pesaran [47] CD, and Baltagi et al. [48] biased-corrected scaled LM.

The Breusch and Pagan [46] model of examining the cross-sectional dependence among the panel data is:

5 $${CD}_{BP}= {\sum }_{i=1}^{N-1}{\sum }_{j=i+1}^{N}{\widehat{{p}_{ij}}}^{2}$$

Pesaran [47] developed the LM statistics to address the limitations of the cure from the above model as:

6 $${CD}_{LM}= \sqrt{\frac{1}{N(N-1)}} {\sum }_{i=1}^{N-1}{\sum }_{j=i+1}^{N}({\widehat{{p}_{ij}}}^{2}-1)$$

If the cross-sectional size is greater than the time dimension, Pesaran [47] recommends the below test statistic:

7 $$CD= \sqrt{\frac{2T}{N(N-1)}} {\sum }_{i=1}^{N-1}{\sum }_{j=i+1}^{N}{\widehat{{p}_{ij}}}^{2}$$

Baltagi et al. [48] developed the simple asymptotic bias correction model, which is:

8 $${CD}_{BC}= \sqrt{\frac{1}{N(N-1)}} {\sum }_{i=1}^{N-1}{\sum }_{j=i+1}^{N}({\widehat{{p}_{ij}}}^{2}-1)-\frac{N}{2(T-1)}$$

where $\widehat{{p}_{ij}}$ specifies a correlation among the errors. In this test, the null hypothesis is H_0_: denotes no cross-sectional dependence and the alternative hypothesis is H_1_: prevalence of cross-sectional dependence.

To make an efficient and robust estimation of the fixed effect model, the model should be homoskedastic with no autocorrelation. If the model suffers from heteroskedasticity, the estimation may be consistent but inefficient [49]. To detect the heteroskedasticity the modified Wald test for group wise heteroskedasticity is performed [50, 71]. Similarly, the presence of of autocorrelation is identified with the aid of Wooldridge [51] auto correlation test for panel data [52].

To overcome the complications of panel data estimation arose due to cross-sectional dependence, heteroskedasticity, and autocorrelation both the panel corrected standard error (PCSE) and the feasible generalized least square (FGLS) models are considered to be best and most efficient. So, the difficulty created due to the panel nature of data, the PCSE, is the path finder [53]. Alternatively, the FGLS model is also able to overcome autocorrelation, heteroskedasticity, and cross-sectional dependence of the estimation [54]. Both the PCSE and FGLS methods are efficient and effective in addressing the heteroskedasticty, autocorrelation and outlier estimates [55–57, 73–76].

To observe the causality between the studied variables, pair-wise Granger [58] causality of stacked test (common coefficients) is employed, where three outcomes are revealed as one-way causality, two-way causality, and no causality. The pair-wise Granger causality equations for the panel data can be written as [59, 60].

9 $${Y}_{i,t}= {A}_{0,i}+{A}_{1,i}{Y}_{i,t-1}+\dots \dots + {A}_{k,i}{Y}_{i,t-1}+ {B}_{1,i}{X}_{i,t-1}+{\Omega }_{i,t}$$

10 $${X}_{i,t}= {A}_{0,i}+{A}_{1,i}{X}_{i,t-1}+\dots \dots + {A}_{k,i}{X}_{i,t-1}+ {B}_{1,i}{Y}_{i,t-1}+{\Omega }_{i,t}$$

where, t indicates the time period dimension of the panel, and i shows the cross-sectional dimension.

The stacked causality test considers all the coefficients are the same across all cross sections as common coefficients [59, 60]. This can be portrayed as:

11 $${A}_{0,i}={A}_{0,j}, {A}_{1,i}={A}_{1,j} ,\dots \dots \dots .,{A}_{k,i}={A}_{k,j},\forall i,j$$

12 $${B}_{0,i}={B}_{0,j}, {B}_{1,i}={B}_{1,j} ,\dots \dots \dots .,{B}_{k,i}={A}_{k,j},\forall i,j$$

Hence the decision rule is H0: Y does not Granger causes X, and H1: Y Granger causes X.

## Results

### Descriptive statistics

The results of the descriptive statistics are shown in Table 1. The mean, median, maximum, minimum, standard deviation, skewness, kurtosis, Jarque–Bera, probability, sum, sum square deviation outcomes of our studied variables are presented, and denotes the robustness and consistency of the estimation.

### Cross-sectional dependence test results

The values of cross-sectional dependence tests and their probabilities are presented in Table 2. All the outcomes have confirmed the significance in all four tests. The coefficients of the variables and their corresponding probability validated our rejection of the null hypothesis of cross-sectional independence. Therefore, Table 2 confirmed our use of cross-sectional dependence of the considered variables.

**Table 1:** Descriptive statistics

	LNFLE	LNFAM	LNAEL	LNFEDS	LNPUH	LNGDP	LNIMM	LNURB
Mean	4.267461	4.881569	4.309613	4.168679	3.008149	24.80490	4.455024	3.491938
Median	4.258905	4.962336	4.364889	4.250048	2.729451	25.07671	4.499810	3.505795
Maximum	4.390788	5.630968	4.605170	4.817100	6.691405	28.62914	4.595120	4.351941
Minimum	4.142976	4.026512	3.563369	3.201299	− 0.614827	20.07097	4.025352	2.656055
Std. Dev	0.067726	0.377061	0.266735	0.400890	1.726997	1.948057	0.148979	0.418792
Skewness	0.109554	− 0.471402	− 0.822261	− 0.611920	0.410460	− 0.341245	− 1.301273	0.130810
Kurtosis	1.805678	2.458394	2.802249	2.383728	2.608485	2.665012	3.954477	2.754632
Jarque–Bera	10.44376	8.374040	19.43354	13.29951	5.859286	4.094235	54.43028	0.911275
Probability	0.005397	0.015191	0.000060	0.001294	0.053416	0.129107	0.000000	0.634044
Sum	725.4684	829.8666	732.6342	708.6754	511.3853	4216.834	757.3542	593.6295
Sum Sq. Dev	0.775178	24.02752	12.02395	27.16040	504.0455	641.3423	3.750913	29.64029
Observations	170	170	170	170	170	170	170	170

All the variables are converted into the natural logarithm form

**Table 2:** Cross-sectional dependence test results

Variables	Breusch–Pagan LM	Pesaran scaled LM	Bias-corrected scaled LM	Pesaran CD
LNFLE	748.844*** (0.000)	74.192*** (0.000)	73.879*** (0.000)	27.362*** (0.000)
LNFAM	592.784*** (0.000)	57.741*** (0.000)	57.429*** (0.000)	22.898*** (0.000)
LNAEL	433.194*** (0.000)	40.919*** (0.000)	40.607*** (0.000)	18.470*** (0.000)
LNFED	510.302*** (0.000)	49.047*** (0.000)	48.735*** (0.000)	21.893*** (0.000)
LNPUH	630.025*** (0.000)	61.667*** (0.000)	61.355*** (0.000)	24.956*** (0.000)
LNGDP	680.909*** (0.000)	67.031*** (0.000)	66.718*** (0.000)	25.981*** (0.000)
LNIMM	209.667*** (0.000)	17.357*** (0.000)	17.045*** (0.000)	5.621*** (0.000)
LNURB	530.505*** (0.000)	51.177*** (0.000)	50.864*** (0.000)	20.728*** (0.000)

***Denotes significance at 1% level. Figures in the parentheses are probabilities

**Table 3:** The results of heteroscedasticity and autocorrelation Test

Model	LNFLE		LNFAM		Existence
Test	Test statistic	*p* value	Test statistic	*p* value	
Modified Wald test for group wise heteroskedasticity	χ^2^ = 72.22	0.000	χ^2^ = 2128.93	0.000	Yes
Wooldridge test for autocorrelation in panel data	F-statistic = 351.239	0.000	F-statistic = 14.621	0.004	Yes

### Heteroskedasticity and autocorrelation results

Table 3 displays the presence of heteroskedasticity and autocorrelation in the model. From the results of chi-square and F-statistics along with their probability values we observe that there is existence of heteroskedasticity and autocorrelation among the panel data of both female life expectancy at birth and female child mortality models.

### The results of PCSE regression

After confirming cross-sectional dependence, heteroskedasticity and autocorrelation the (PCSE) model is estimated and the results of both models are reported in Table 4.

In case of female life expectancy at birth, from Table 4, it is observed that the coefficients of access to electricity, female education, public health expenditure, GDP, immunization rate, and urbanization rate are 0.017, 0.041, 0.007, 0.009, 0.028, and 0.041, respectively, which all are positive and are statistically significant at 1% level, except the urbanization rate. These imply that 1% increase of access to electricity, female education rate, public health expenditure, GDP, immunization rate, and urbanization rate will increase female life expectancy at birth by 0.017%, 0.041%, 0.007%, 0.009%, 0.028%, and 0.041%, respectively.

These findings show that the access to electricity, female education rate, public health expenditure, GDP, immunization rate, and urbanization rate improves the female life expectancy at birth, which indicates their better health status in the studied regions.

In Table 4, for the female adult mortality rate, the coefficients of access to electricity, GDP, and immunization rate are − 0.097, − 0.039, and − 0.117, which all are negative and statistically significant at 5% level. The coefficients of female education, and public health expenditure, − 0.211, and − 0.054, consecutively, which are negative and are statistically significant at 1% level. These results imply that 1% increase of access to electricity, female education rate, public health expenditure, GDP, and immunization rate decrease the female adult mortality rate by 0.097%, 0.211%, 0.054%, 0.039%, and 0.117%, respectively. The coefficient of urbanization is 0.108 which is positive but statistically insignificant. From the results, it is found that, access to electricity, female education rate, public health expenditure, GDP, and immunization rate reduce the female adult mortality rate, which is desirable for explaining better female health status in the considered regions.

**Table 4:** Panel corrected standard error (PCSE) model results

Variables	PCSE regression (LNFLE case)	PCSE regression (LNFAM case)
LNAEL	0.017*** (3.65)	− 0.097** (− 2.04)
LNFED	0.041*** (6.96)	− 0.211*** (− 4.01)
LNPUH	0.007*** (4.92)	− 0.054*** (− 3.63)
LNGDP	0.009*** (5.04)	− 0.039** (− 2.42)
LNIMM	0.028*** (4.85)	− 0.117** (− 2.01)
LNURB	0.041*** (3.02)	0.108 (0.98)
_Constant	3.519*** (65.84)	7.478*** (17.75)
R-squared	0.9996	0.9843
Wald chi^2^	446.94	105.66
Probability	0.000	0.000
N	170	170

*** and ** denote significance at 1% and 5% levels respectively. Figures in the parentheses are z-statistics

### Robustness check: the results of FGLS regression

The robustness of the results obtained from the PCSE model is confirmed by the FGLS model as shown in Table 5. The Table 5 confirms that access to electricity, female education rate, public health expenditure, GDP, immunization rate, and urbanization rate positively and significantly affect female life expectancy. On the other hand, access to electricity, female education rate, public health expenditure, GDP, and immunization rate have a negative effect on the female adult mortality rate, whereas the urbanization rate has an insignificant impact on the female adult mortality rate.

### Pairwise granger causality tests results

The result of pair-wise Granger causality test is depicted in Table 6, where the results are presented by F-statistic and corresponding probability. We achieve one-way causality of female life expectancy with access to electricity, female education rate, GDP, immunization rate, and urbanization rate. Similarly, we also obtained one-way causality of the female adult mortality rate with access to electricity, female education rate, and immunization rate.

## Discussion

The outcomes of Tables 5 and 6 display the key determining factors of female life expectancy at birth and female adult mortality rate. We have found that access to electricity positively affects female life expectancy and negatively affects the female adult mortality rate due to the convenience of the operation of modern medical instruments and preservation of valuable medicines, drugs, and vaccination and receiving health related information from public media. Thus, more access to electricity enhances life extending facilities enough to reduce mortality rate and increase the life expectancy of female. These outcomes are in the line with the findings of Wang [6], Chen et al. [7], Adair-Rohani et al. [11], Irwin et al. [12], and Hernández [14]. A higher female education rate increases life expectancy and decreases the female adult mortality rate because education creates and enhances awareness and consciousness among females to consume nutritious food, to maintain a healthy life for them and for their children. Moreover, the educated female can acquire better health related information to stay healthy and lead a safe life. This result is consistent with those of Rahman and Alam [8], McAlister and Baskett [16], Keats [19], and Kanmiki et al. [20]. Public health expenditure has significant and positive effect on female life expectancy, and negative impact on the female adult mortality rate, as it promotes different public hospitals, free of cost medicines, and lower cost medical facilities for people. Additionally, the more public health expenditure arranges better medical facilities (e.g. more hospitals, healthcare workers, medicines, etc.) for the poor and vulnerable portion of population including female. This outcome is similar to the findings of Rahman et al. [9], Novignon and Lawanson [10], Nicholas et al. [23], Ahmad and Hasan [24], and Behera and Dash [72]. The immunization rate also improves female life expectancy and reduces female adult mortality. Vaccination creates herd immunity against infectious diseases, as a result children and adults become less affected by various types of infectious diseases, signifying an improved health status for females. Therefore, the availability of vaccination to the mass level including female can ensure better immunity that lengthens female life expectancy and reduces mortality rate This outcome is consistent with the results obtained by Pezzotti et al. [32], Brisson et al. [33], Owais et al. [34], and Rodrigues and Plotkin [61], considering health outcomes. The per capita GDP positively influences female life expectancy and negatively influences female adult mortality, because economic growth enables governments to spend more in the health sector, and ensures more medical facilities and better living standards for people, which significantly improves their health status. The higher growth rate makes family affluent; household head can spend more for the better health of family members including females. This finding is similar to those of Rahman and Alam [8], Rahman et al. [9], Wang et al. [25], Mahyar [35], and Shahbaz et al. [36]. The urbanization rate has a positive effect on female life expectancy but an insignificant effect on the female adult mortality rate because of improved urban health facilities, access to information, better living standards, better employment resources and higher incomes for all people. The urban people also become more health conscious due to the availability of more urban facilities. Furthermore, in urban areas, most of the females are employed and can afford better health facilities and better food that enhance their life expectancy. This result is consistent with the results of Wang [39], Amouzou et al. [40] and Panahi and Aleemran [41], but not consistent with the results of Rahman and Alam [8], Adediran et al. [42], and Torres et al. [62].

**Table 5:** Feasible generalized least square (FGLS) model results

Variables	FGLS regression (LNFLE case)	FGLS regression (LNFAM case)
LNAEL	0.010*** (2.82)	− 0.089** (− 2.41)
LNFED	0.024*** (5.14)	− 0.138*** (− 3.79)
LNPUH	0.004*** (3.31)	− 0.023** (− 2.34)
LNGDP	0.010*** (6.68)	− 0.060*** (− 5.04)
LNIMM	0.014*** (3.07)	− 0.090** (− 2.15)
LNURB	0.126*** (9.06)	0.093 (1.27)
_Constant	3.334*** (70.12)	7.570*** (22.96)
Wald chi^2^	634.86	124.60
Probability	0.000	0.000
N	170	170

*** and ** denote significance at 1% and 5% levels respectively. Figures in the parentheses are z-statistics

**Table 6:** Causality test results

Null Hypothesis:	F-Stat	Prob	Decision
LNFLE case			
LNAEL does not cause LNFLE	0.010	0.991	LNFLE → LNAEL (one-way causality)
LNFLE does not cause LNAEL	7.788***	0.001	
LNFED does not cause LNFLE	0.162	0.850	LNFLE → LNFED (one-way causality)
LNFLE does not cause LNFED	4.654**	0.011	
LNPUH does not cause LNFLE	0.775	0.463	No causality
LNFLE does not cause LNPUH	0.199	0.819	
LNGDP does not cause LNFLE	2.512*	0.085	LNGDP → LNFLE (one-way causality)
LNFLE does not cause LNGDP	1.529	0.220	
LNIMM does not cause LNFLE	0.610	0.545	LNFLE → LNIMM (one-way causality)
LNFLE does not cause LNIMM	2.948*	0.056	
LNURB does not cause LNFLE	22.828***	0.000	LNURB → LNFLE (one-way causality)
LNFLE does not cause LNURB	1.769	0.174	
LNFAM case			
LNAEL does not cause LNFAM	0.189	0.828	LNFAM → LNAEL (one-way causality)
LNFAM does not cause LNAEL	5.038***	0.008	
LNFED does not cause LNFAM	0.162	0.851	LNFAM → LNFED (one-way causality)
LNFAM does not cause LNFED	2.356*	0.098	
LNPUH does not cause LNFAM	0.258	0.773	No causality
LNFAM does not cause LNPUH	0.683	0.507	
LNGDP does not cause LNFAM	0.275	0.759	No causality
LNFAM does not cause LNGDP	1.601	0.205	
LNIMM does not cause LNFAM	0.162	0.851	LNFAM → LNIMM (one-way causality)
LNFAM does not cause LNIMM	3.826**	0.024	
LNURB does not cause LNFAM	1.925	0.150	No causality
LNFAM does not cause LNURB	0.330	0.719	

***, ** and * denote significance level at 1%, 5%, and 10%, respectively

## Conclusion and policy implications

This paper has explored the nexus of access to electricity, female education, and public health expenditure with female health outcomes in the SAARC-ASEAN countries. Using the data of 2002–2018, and applying the cross-sectional dependence test, Modified Wald test, Wooldridge test, the PCSE model and the FGLS model, and the pair-wise Granger causality test, robust results have been obtained. Access to electricity, female education rate, public health expenditure, economic growth, and immunization rate have a positive effect on female life expectancy at birth, and a negative effect on the female adult mortality rate. The urbanization rate has a significant positive impact on female life expectancy at birth but an insignificant impact on the female adult mortality rate. A one-way causal relationship between the variables is also noted. All the results are logical and generate important milestones for the health sector. These results may be said to apply, not only for the regions studied but globally.

The important policy implications of the study are: the health status of females should be improved and protected by formulating effective policies on access to electricity, female education, public health expenditure, immunization, economic growth, and urbanization. In this context the following specific recommendations should be prioritized:

(i) *Ensuring more access to electricity:* More electricity access plays role in increasing female life expectancy and reducing female mortality rate in the studied region. Electricity facility also helps females to be health conscious via electricity run devices and media by and also helps to take proper medical treatment with modern electricity led medications. It also helps female to get employment and thus discourages child marriages. Use of electricity facilitates easy access to print and electronic media that increases female awareness, which plays an important role in reducing early marriage related death and increasing life expectancy. For this reason, an effective and efficient policy formulation to ensure more electricity access should be formulated that will be conducive to female health.
(ii) *Spreading more female education:* More education of female ensures more life expectancy and lower female mortality rate[19, 20]). Thus, all types of barriers of education for female should be eradicated and priority should be given to a female friendly environment for spreading more female education, so that, among others, they will be more aware of about health issues. For this reason more female educational institutions, financial facilities by offering various scholarships, and a better educational environment are required which urge dynamic policy efforts.
(iii) *Greater public health expenditure:* Our findings of public health expenditure on female life expectancy and female mortality rate plausible. More public health expenditure offers more medical facilities at no cost or minimum costs As a result, people including female can easily enjoy required health facilities which reduces mortality and lengthens female life expectancy.. Therefore, the governments of these countries should increase budget allocation for public health with priority for females’ health as women has unique health issues [77]. In this regard, supplying more doctors specializing in female health, establishing more community clinics at the foundational level, more subsidies for medicines, and providing modern and improved medical equipment are essential.
(iv) *Larger coverage on immunization*: As immunization builds herd immunity against different types of infectious diseases, a greater coverage of immunization may help females to achieve better health outcomes [32, 33]. Necessary arrangements should be made in such a way that all people including female may get vaccinated. In this case, secular, unbiased, and gender friendly immunization policies should be formulated.
(v) *Sustainable economic growth policy* Our findings of economic growth on health outcomes are consistent too because economic growth brings amenities and ensures modern types of different facilities for better health, which increases female life expectancy and declines female mortality rate. A sustainable and effective economic growth policy can ensure modern health facilities for females. Therefore, effective, efficient, updated, and health-focused economic growth policies should be pioneering across the regions for making sustainable future for ensuring better female health status.
(vi) *Planned urbanization* As urbanization increases female life expectancy, the more panned urban facilities for female should also be ensured. Because unplanned urbanization may create detrimental consequences on human health through creating pollution, unhygienic water and sanitation facilities, congestion, and various socio-cultural mal-adjustments like slums [8, 62]. A proficient, green, and sustainable well-organized and well-planned urbanization policy should be undertaken to ensure better health status for all people, but particularly for females.

## Data Availability

Data used in this study are collected from the World Development Indicator (WDI, 2020) of the World Bank. The complete structured data set used for the study may be supplied by the corresponding author on reasonable request.

## Footnotes

1 The SAARC-ASEAN countries are Bangladesh, Bhutan, India, Nepal, Pakistan, Sri Lanka, Brunei Darussalam, Myanmar, Philippines, and Thailand.
